# Characterizing On-Road CO_2_ and NO_x_ Emissions of LNG and Diesel Container Trucks Using Portable Emission Measurement System

**DOI:** 10.3390/s26061868

**Published:** 2026-03-16

**Authors:** Hongmei Zhao, Zhaowen Han, Lijun Cheng, Yuxuan Lyu, Tian Luo

**Affiliations:** 1 College of Transport & Communications, Shanghai Maritime University, Shanghai 201306, China; hmzhao@shmtu.edu.cn (H.Z.); 202530610123@stu.shmtu.edu.cn (Z.H.); 202310621039@stu.shmtu.edu.cn (Y.L.); 2 School of Economics & Management, Shanghai Maritime University, Shanghai 201306, China; ljcheng@shmtu.edu.cn; 3 Alibaba Business School, Hangzhou Normal University, Hangzhou 311121, China

**Keywords:** LNG container truck, portable emission measurement system (PEMS), real-world emission, machine learning

## Abstract

Heavy-duty vehicles (HDVs) are major greenhouse gas emitters, and liquefied natural gas (LNG)-powered HDVs have emerged as a promising low-carbon alternative. However, their real-world emission performance and mitigation potential remain insufficiently studied, necessitating the characterization of LNG container trucks’ on-road CO_2_ emissions via advanced sensing technologies. To characterize HDVs’ emission characteristics, real-driving emissions from China VI LNG and diesel-powered container trucks were measured employing portable emissions measurement systems (PEMS). The results reveal that high CO_2_ emissions predominantly occur during low- to medium-speed acceleration and at speeds above 40 km/h with an acceleration exceeding 0.3 m/s^2^ on highways, whereas emissions on port roads are more dispersed. A third-degree polynomial function fits emissions well with vehicle-specific power (VSP). Engine parameters mainly influence CO_2_ emissions for LNG trucks, while VSP and acceleration significantly impact diesel trucks. The Random Forest model achieves superior prediction accuracy, particularly in highway scenarios, and significantly better CO_2_ forecasting for LNG-powered trucks. These findings validate the effectiveness of PEMS-based sensing in characterizing low-carbon HDVs’ real-world emissions. The integration of multi-source sensor data and machine learning also provides a reference for intelligent sensing in transportation environmental monitoring.

## 1. Introduction

Diesel-powered container trucks have long dominated China’s road freight sector, accounting for about 85% of the total container truck fleet (estimated at 1.2 million units in 2024) [1,2]. Their prevalence stems from mature infrastructure, high torque output suitable for heavy loads, and lower upfront costs. However, stricter China VI regulations (GB 17691-2018, China VI a/b implemented in 2019/2023) and rising diesel prices have accelerated fleet renewal [3]. Since 2020, over 400,000 old China III/IV diesel container trucks have been phased out, with China VI diesel models now representing about 60% of the operational fleet [4]. Unlike previous bench test only standards, China VI mandates both laboratory and real-world emission compliance, strictly regulates On-Board Diagnostic (OBD), after-treatment durability and in-use monitoring, and tightens limits for CO, NO_x_, particulate matter (PM), non-methane hydrocarbons (NMHC_S_), and particle number (NO_x_/PM reduced by 77%/67% vs. China V). Notably, methane slip is not directly regulated but indirectly constrained via total hydrocarbons/NMHC_S_ limits, though its control as a potent greenhouse gas is an inevitable future trend. Despite this, diesel trucks remain the primary choice for long-haul highway transport (e.g., between ports and inland logistics hubs) due to their extensive refueling network.

LNG container trucks have emerged as a key low-carbon substitute for diesel in China, driven by policy support and environmental advantages (e.g., 20–30% lower CO_2_ emissions and 80–90% lower NO_x_ emissions compared to China V diesel trucks) [2]. As of 2024, LNG container trucks hold a 15% share of the total container truck market, with annual growth rates exceeding 25% since 2020 [1]. Their adoption is concentrated in coastal regions such as Shanghai, Guangdong, and Shandong areas home to China’s busiest ports (for example, Shanghai Port handled approximately 47 million Twenty-foot Equivalent Units in 2024, ranking first globally). These regions are likewise characterized by well-developed LNG bunkering infrastructure, with the national inventory of LNG refueling stations exceeding 3000, representing a twofold increase relative to 2019 [2,5].

CO_2_ is a primary greenhouse gas produced by the complete combustion of hydrocarbon fuels such as diesel and LNG, whereas incomplete combustion due to oxygen deficiency or inadequate fuel–air mixing typically results in CO emissions. NO_x_, consisting mainly of NO and NO_2_, is predominantly formed through the thermal NO_x_ mechanism at high in-cylinder temperatures, accompanied by minor contributions from prompt NO_x_ and negligible fuel NO_x_ for LNG engines. PM comprises soot, organic compounds, sulfates, and metal oxides, mostly originating from the incomplete combustion of diesel fuel. With the surge in vehicle ownership and the rapid advancement of the transportation sector, the emissions of CO_2_, CO, and NO_x_ from vehicles have emerged as the primary contributors to urban air pollution [6], posing a serious threat to residents’ health. Heavy-duty vehicles, particularly container trucks, play a crucial role in road transportation. Although their ownership is relatively small, their CO_2_ and NO_x_ emissions account for a relatively large proportion of mobile sources [1,7,8], posing a major challenge to the green development of the transportation industry [4].

Research has shown that laboratory test cycles fail to reflect the real on-road emission levels of HDVs [2]. In real-world urban driving environments, the engine speed and torque of heavy-duty vehicles exhibit substantial fluctuations, attributed to the variability of driving behaviors and traffic scenarios. In contrast to normal or moderate driving styles, aggressive driving is characterized by a lower idling proportion and more sudden load increases, which tend to induce sharp spikes in NO_x_ emissions [9]. Weiss et al. (2011) employed the PEMS to test NO_x_ emissions from light-duty vehicles and revealed that Euro 5 diesel vehicles emitted NO_x_ at levels 2.3 to 4.2 times higher than the regulatory standards, and their CO_2_ emissions were 12% to 30% greater than those obtained in laboratory tests [10]. Wang et al. (2021) demonstrated that emission factors for fully loaded HDVs in low-speed driving scenarios were elevated by 96.2% (CO_2_), 47.9% (CO), and 65.2% (NO_x_) in comparison to the unloaded operation [11]. Wang et al. (2018) found that the specific emissions of CO and NO_x_ from China VI diesel vehicles increased markedly with altitude [12], a pattern similarly reported in Chen et al. (2023) [13]. Furthermore, the precision of PEMS measurements is essential for the credibility of the measured on-road emissions. On-road emissions from light-duty gasoline vehicles, diesel vehicles, and hybrid buses have demonstrated that PEMS is highly credible and accurate in quantifying vehicle pollutant emissions [14,15].

In response to these gaps, China VI requires heavy-duty vehicles to meet emission limits in both laboratory bench tests and real-road driving conditions (covering highway, urban, and suburban scenarios), and also strictly regulates the OBD system, emission after-treatment system durability, and in-use compliance monitoring. Since the promulgation of China VI in 2018, the main progress in heavy-duty vehicle emission control has focused on full nationwide implementation, strengthened enforcement, mandatory real-driving emission tests, expanded remote OBD monitoring, and policy support for new energy and clean fuel vehicles, rather than fundamental revisions to the emission limits themselves. This policy shift reflects global efforts to align emission standards with real-world driving conditions. A PEMS study conducted by Grigoratos et al. (2019) on Euro VI HDVs in real driving conditions showed that their CO, NO_x_, and particulate number emissions were consistently lower than the maximum values allowed under the Euro VI Standard [16]. Notably, while international research has focused on NO_x_ and particulate matter control for HDVs [17,18,19], greenhouse gas (GHG) emissions like CO_2_ have received less attention, partly due to historical limitations in on-board measurement technologies. This neglect is particularly acute for China VI heavy-duty LNG vehicles, where empirical CO_2_ data under diverse operational conditions (e.g., highway vs. port roads) are scarce. This research gap is critical given China’s commitment to carbon neutrality and the transportation sector’s role in achieving this goal.

In summary, China VI LNG and diesel container trucks are not only central to China’s efforts to reduce transportation emissions but also serve as a global case study for balancing freight efficiency, regulatory compliance, and low-carbon transition. Given the significant gap between the real-world emissions of motor vehicles and the results of laboratory test cycles, there is an urgent need for more accurate and comprehensive on-road emission testing and analysis. Focusing on this scientific gap, this study applies PEMS measurements to evaluate real-driving emissions of CO_2_ and NO_x_ from China VI LNG and diesel container trucks. The results of this study are expected to offer essential data support for accurately determining container truck emission factors, improving emission inventory calculations, and guiding the formulation of more effective emission control policies and future emission limits for real-world GHG emissions.

Against the backdrop of China’s carbon neutrality target and the ongoing revision of vehicle emission regulations, this study will bridge the gap between laboratory testing and real-world emissions. Our findings will serve as a foundation for formulating evidence-based policies to mitigate GHG emissions from HDVs, aligning with global efforts to address climate change through transportation sector decarbonization. The principal contributions of this research can be outlined as follows:

(1) By quantifying real-world emissions under typical operational conditions (highways and port roads), this study provides essential data to refine China’s emission inventory and support the development of GHG emission factors for HDVs. This is particularly timely as China seeks to integrate CO_2_ control into future vehicle emission standards.

(2) The comparison between LNG and diesel container trucks offers insights into the environmental performance of alternative fuels, informing policies to promote clean energy adoption in heavy-duty transportation.

(3) Machine learning approaches such as the Random Forest model improve the precision of real-world emission evaluations, facilitating more effective regulatory monitoring and compliance verification.

## 2. Materials and Method

### 2.1. Equipment

**Figure 1:**
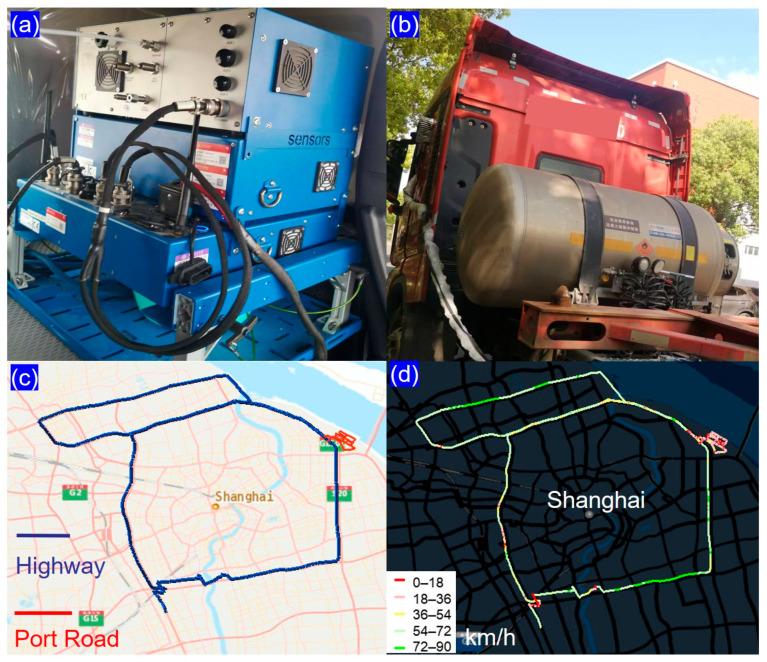
Experimental equipment, vehicles, routes, and driving speeds based on PEMS. (**a**) Experimental equipment; (**b**) Test Vehicles; (**c**) Test route; (**d**) The vehicle speed during the experiment.

In this study, a SEMTECH-DS+ portable emission measurement system manufactured by Sensors, Inc., located in Saline, MI, USA, was employed to acquire real-time instantaneous and cumulative emission data of the tested vehicles under actual driving conditions (Figure 1a). The equipment draws power from two external lithium-ion batteries with high energy density, arranged in a parallel configuration. The integrated measurement system comprised multiple components: (1) a gas analyzer for CO_2_ and NO_x_ quantification, (2) an exhaust flow meter monitoring mass flow rate, temperature, and pressure, (3) an OBD interface capturing engine parameters (speed, torque, and power) and vehicle speed, (4) meteorological sensors recording ambient conditions (temperature, humidity, altitude, barometric pressure), and (5) a global positioning system unit logging positional data (latitude, longitude, and altitude) and velocity at 1 Hz frequency. CO_2_ concentrations were quantified using non-dispersive infrared spectroscopy, while NO_x_ levels were determined through non-dispersive ultraviolet absorption techniques. Ambient meteorological conditions, particularly temperature and relative humidity, were monitored using a dedicated weather probe.

The quality assurance and quality control procedures were rigorously implemented before each experimental run, beginning with a mandatory 60 min preheating of the SEMTECH-GAS analyzer to ensure optimal operating conditions. Following thermal stabilization, the system underwent a thorough purification process involving the injection of over 3 L of high-purity nitrogen gas to establish accurate zero baselines, remove residual gases, and verify system integrity through leak testing. All instruments were subjected to comprehensive flow rate and concentration verification checks prior to data collection. For the critical CO_2_ and NO_x_ measurements, daily zero-span calibration procedures were performed, with span calibrations conducted immediately after successful zeroing to maintain measurement accuracy throughout the experimental campaign. These standardized protocols ensured the reliability and precision of all emission data collected during real-world driving conditions.

### 2.2. Test Vehicles and Route

The tested container trucks meeting China VI emission standards for diesel and LNG were selected in this study, and their detailed technical specifications are listed in Table 1. Both test vehicles were commercially leased from a major freight company in Shanghai and represent mainstream models widely used in coastal port logistics in China (Figure 1b). The LNG truck was manufactured in 2023 and the diesel truck in 2020, and both were in sound operational conditions with no mechanical or emission control system abnormalities, ensuring high representativeness for in-service fleets. The LNG engine is not a simple retrofit of a diesel engine, but a purpose-built design derived from a mature diesel platform with high component commonality to guarantee reliability and cost-effectiveness, while incorporating dedicated optimizations for natural gas fuel, including a spark ignition system, enhanced thermal management, and a stoichiometric combustion system with high-pressure exhaust gas recirculation and a three-way catalyst (TWC) to satisfy China VI requirements. In contrast, the diesel truck employs lean-burn combustion integrated with a diesel oxidation catalyst (DOC), diesel particulate filter (DPF), selective catalytic reduction (SCR), and ammonia slip catalyst after-treatment devices. During testing, the curb weights of the LNG and diesel trucks were 7.3 t and 8.87 t, respectively, and both were loaded with a standard empty 40-foot container (approximately 4 t), consistent with typical port logistics payloads. Although the two vehicles could not be tested simultaneously due to limitations of experimental equipment and vehicle leasing arrangements, they followed identical routes and experienced similar traffic conditions. Each test vehicle was equipped with an OBD monitoring system for real-time data acquisition.

The test routes were designed to reflect the typical real-world operating conditions of container trucks serving the Shanghai Port, with a round-trip distance of approximately 160 km per test cycle, primarily consisting of highway and port access roads (Figure 1c). The route consists of 130 km of highways (Shanghai S20 Outer Ring Expressway and G1503 Shanghai Ring Expressway), accounting for 81% of the total length. These highways represent typical intercity corridors for port-to-inland logistics. The remaining 30 km (19%) includes port access roads and internal yard roads in Waigaoqiao Port, characterized by low-speed and frequent stop-and-go driving conditions. Construction zones and other atypical road segments were excluded from the test routes, and all tests were performed between 11:00 and 20:00, corresponding to the peak operating hours of port container trucks, thus ensuring the representativeness of the driving conditions. Representative velocity profiles for these routes are illustrated in Figure 1d. Highway driving maintained steady 50 km/h average speed, contrasting with port road operations that averaged 20 km/h (peaking at 40 km/h in limited sections). The real driving emissions tests were conducted on 8–9 May and 14–15 May 2024 from 11:00 to 20:00 local time in Shanghai.

### 2.3. Key Parameters Related to Emissions

Three categories of key parameters—driving patterns, engine performance, and environmental conditions—were adopted to elaborate on their impacts on the emission characteristics of CO_2_ and NO_x_. Specifically, engine performance parameters include engine load (EL), percentage torque (PT), engine speed (ES), and engine power (EP), all of which were collected through the OBD system integrated with the SEMTECH analyzer. Extant research has demonstrated that engine performance parameters can effectively characterize the operating status of vehicle engines and exert a significant influence on CO_2_ and NO_x_ emissions. The driving characteristics include vehicle speed (VS), vehicle acceleration (VR), and VSP. VSP serves as a key parameter for characterizing driving conditions and emissions in motor vehicles. The slope factor is not incorporated in the calculation formula for VSP, as the topographic slope in Shanghai exhibits minimal variation. The formulation is expressed as follows [20]:

(1) $$VSP=av+\frac{A}{m}v+\frac{B}{m}v^{2}+\frac{C}{m}v^{3},$$

VSP, expressed in kilowatts per ton (kW/t), serves as a critical metric for assessing vehicle operating conditions and emission patterns. The calculation incorporates several key parameters: instantaneous vehicle acceleration (*a*, m/s^2^), vehicle speed (*v*, m/s), and three empirically derived coefficients accounting for rolling resistance (*A*, kW·s/m), rotational resistance (*B*, kW·s^2^/m^2^), and aerodynamic drag (*C*, kW·s^3^/m^3^). m refers to the vehicle weight in tons. In this study, the coefficients $\frac{A}{m}$, $\frac{B}{m}$ and $\frac{C}{m}$ used for heavy-duty diesel vehicles with a gross vehicle weight exceeding 12 tons were 0.0857, 0, and 0.000331, respectively [20].

Environmental conditions refer to the ambient on-road factors, including ambient temperature (AT), ambient pressure (AP), and relative humidity (RH). These parameters were selected based on their direct effects on engine combustion processes and the consequent emissions of CO_2_ and NO_x_ [9]. Although these factors are known to influence vehicle emissions [12,13], their contributions for China VI LNG and diesel trucks under port and highway conditions remain understudied. During tests in Shanghai (8–9 May and 14–15 May 2024), pressure was stable (coefficient of variation (CV) = 0.16%), relative humidity varied moderately (CV = 16.13%), and temperature fluctuated slightly (CV = 3.07%). All environmental conditions were within normal ranges for emission analysis.

### 2.4. Models and Performance Evaluation

As a bagging-based ensemble algorithm, Random Forest (RF) constructs numerous decision trees using randomly selected training subsets. The RF model typically achieves high prediction accuracy and has been widely employed in various fields such as economics and materials research [18,21]. The RF model employs either the Gini index or out-of-bag error rate as evaluation metrics to quantify variable importance [21]. In this study, we applied the RF model to determine the relative contribution of various factors, including engine parameters, driving conditions, and external characteristics, to CO_2_ and NO_x_ emissions.

Linear Regression (LR) represents a foundational statistical modeling approach that establishes a linear relationship between a response variable and its explanatory variables. The LR model serves as a basis for more complex regression techniques and is often employed as a benchmark for comparing the performance of other advanced regression algorithms. Bagging Regression (BR) is an ensemble method designed for regression analysis, employing the Bootstrap Aggregating (bagging) principle. By combining predictions from multiple base regression models, BR enhances overall predictive accuracy while mitigating overfitting. Extreme Gradient Boosting (XGB) represents an advanced evolution of Gradient Boosted Regression Trees (GBRT). Unlike traditional GBRT, which relies solely on first-order derivatives, XGB incorporates second-order Taylor expansion of the loss function. The algorithm’s fundamental approach involves iteratively adding decision trees to systematically minimize the difference between predicted and actual values [22]. The k-Nearest Neighbors (KNN) algorithm operates as a non-parametric, instance-based learning method that makes predictions by measuring similarity between feature space observations. For regression applications, KNN calculates a weighted average (or other suitable aggregation) of the target values from the *k* closest training instances, rather than employing classification’s majority voting approach.

To prevent overfitting of the machine learning emission prediction model, the cleaned portable emissions measurement system dataset was randomly divided into 80% training and 20% test subsets without overlap, and five-fold cross-validation was implemented during model training to avoid overfitting associated with fixed data partitioning. Key hyperparameters of the RF model, including the number of decision trees, maximum tree depth, and minimum sample size for node splitting, were optimized via grid search to restrict excessive model complexity. In addition, feature selection was conducted using Spearman correlation analysis, multicollinearity testing, and RF feature importance ranking to reduce feature dimensionality and mitigate overfitting due to dimensionality.

Two primary metrics are commonly used to evaluate the predictive accuracy of forecasting models. The coefficient of determination (R^2^) measures the proportion of variance explained by the model, and the root mean square error (RMSE) assesses the magnitude of prediction deviations. Optimal model performance is indicated by R^2^ values approaching 1 and minimized RMSE values [23,24].

## 3. Results

### 3.1. Impacts of Driving Patterns on CO_2_ and NO_x_ Emissions

#### 3.1.1. Impact of Speed and Acceleration on CO_2_ and NO_x_ Emissions

**Figure 2:**
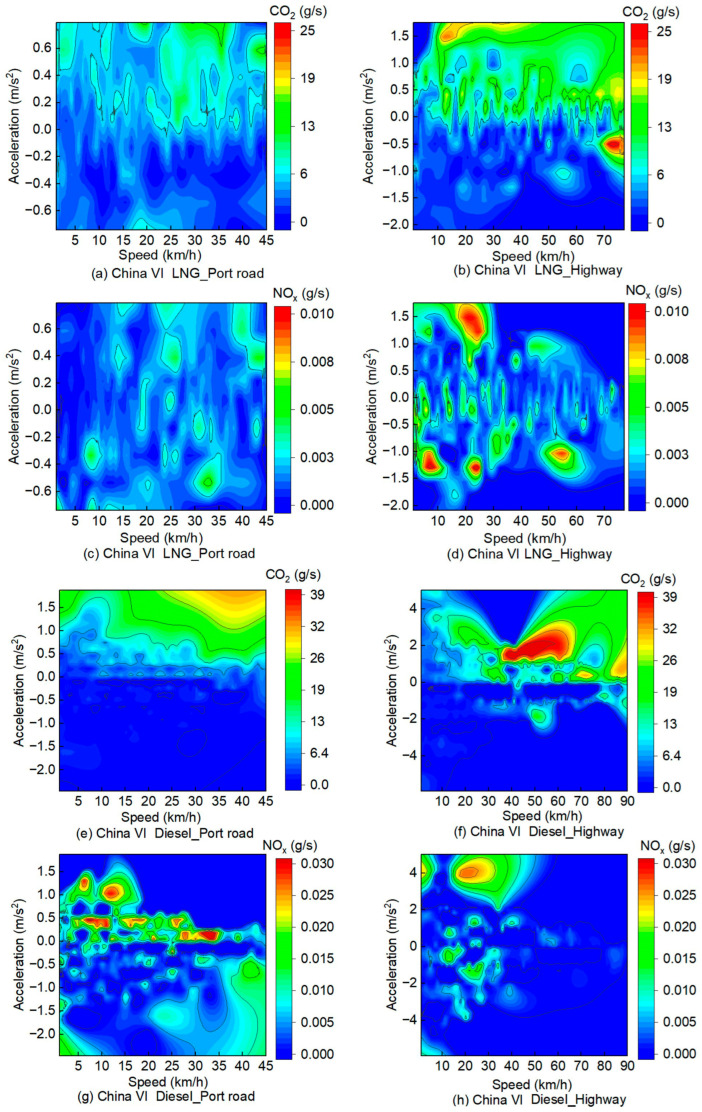
PEMS-measured instantaneous emission rates of CO_2_ and NO_x_ from China VI LNG versus diesel trucks under varied roadway operating conditions. (**a**) China VI LNG_Port road for CO_2_; (**b**) China VI LNG_Highway for CO_2_; (**c**) China VI LNG_Port road for NO_x_; (**d**) China VI LNG_Highway for NO_x_; (**e**) China VI Diesel_Port road for CO_2_; (**f**) China VI Diesel_Highway for CO_2_; (**g**) China VI Diesel_Port road for NO_x_; (**h**) China VI Diesel_Highway for NO_x_.

Instantaneous emission rates of both CO_2_ and NO_x_, measured via PEMS for China VI LNG and diesel container trucks operating under different roadway classifications, are depicted in Figure 2. All vehicle speed-acceleration interval data are continuous, and the results indicate that CO_2_ emissions from LNG container trucks increase gradually with rising speed, though the growth rate slows down progressively. This suggests that while total emissions continue to rise at higher speeds, the rate of emissions per unit of time decreases. LNG container trucks exhibit lower NO_x_ emissions when traveling on highways, because highway driving conditions are generally more stable characterized by smaller speed fluctuations and smoother vehicle operation. Such conditions optimize engine combustion processes, thereby reducing NO_x_ formation [25].

As depicted in Figure 2e–h, CO_2_ emissions from diesel trucks also rise with increasing speed, but the growth rate is more pronounced, as diesel engines typically exhibit lower combustion efficiency at lower speeds. Diesel trucks emit higher NO_x_ concentrations on port roads, primarily because heavy traffic and frequent stop-start operations on such roads lead to unstable combustion in diesel engines, thereby facilitating greater NO_x_ formation.

Additionally, owing to the clean energy characteristics of LNG container trucks, their NO_x_ emissions are lower than those of diesel container trucks, particularly during rapid acceleration. Acceleration has a more pronounced impact on diesel truck emissions. During rapid acceleration conditions, the engine’s elevated fuel demand for power generation triggers a steep increase in CO_2_ output, while combustion instability simultaneously exacerbates NO_x_ formation through thermal mechanisms [26]. In summary, vehicle type and road conditions are key factors affecting vehicle emission performance. By optimizing vehicle design, enhancing emission control systems, and improving road conditions, it is feasible to effectively reduce vehicle emissions and thus improve air quality.

#### 3.1.2. Impact of VSP on CO_2_ and NO_x_ Instantaneous Emission Rates

**Figure 3:**
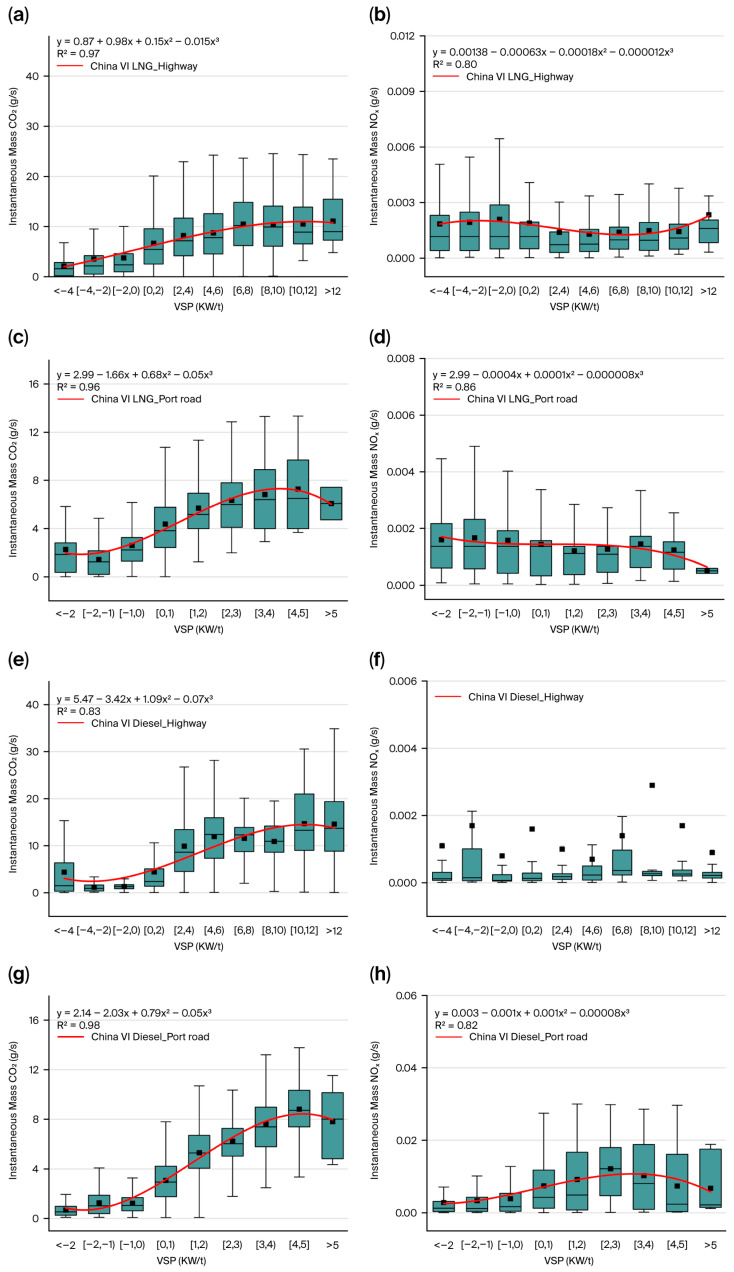
CO_2_ and NO_x_ emissions of China VI container trucks under driving condition bins. (**a**) China VI LNG_Highway for CO_2_; (**b**) China VI LNG_Highway for NO_x_; (**c**) China VI LNG_Port road for CO_2_; (**d**) China VI LNG_Port road for NO_x_; (**e**) China VI Diesel_Highway for CO_2_; (**f**) China VI Diesel_Highway for NO_x_; (**g**) China VI Diesel_Port road for CO_2_; (**h**) China VI Diesel_Port road for NO_x_.

Figure 3 illustrates the pollutant emission rates across different VSP ranges. It is observed that CO_2_ emissions from LNG container trucks increase gradually with rising VSP, though the growth rate decelerates progressively. This suggests that at lower VSPs (i.e., lower speeds or accelerations), the increase in CO_2_ emissions is more significant. In contrast, at a higher VSP, while emissions still rise, the rate of increase slows down. This phenomenon is linked to the improved efficiency of LNG engines under higher loads. Compared to diesel container trucks, LNG container trucks generally exhibit lower NO_x_ emissions. During VSP fluctuations, NO_x_ emissions from LNG container trucks remain relatively stable with low overall levels, due to LNG’s clean energy nature, which generates less NO_x_ during combustion [27].

The CO_2_ emissions of diesel trucks also increase with rising VSP, with a more pronounced growth rate. This reflects the lower combustion efficiency of diesel engines under lower loads, which results in a faster increase in CO_2_ emissions. At a higher VSP, while emissions continue to rise, the growth rate slows compared to a lower VSP due to improved engine efficiency. The NO_x_ emissions of diesel container trucks are significantly affected by VSP.

Both types of container trucks show an increase in CO_2_ emissions as VSP rises. However, there is a difference in NO_x_ emissions. LNG trucks exhibit significantly lower NO_x_ emissions than those diesel trucks, and their NO_x_ emissions are less affected by VSP fluctuations. This is mainly because LNG, as a clean energy source, produces less NO_x_ during combustion [9]. In contrast, diesel trucks’ NO_x_ emissions are highly influenced by VSP, especially during large load variations in port road. It is worth noting that freeway operations feature stable medium-to-high vehicle speeds, sustaining exhaust temperatures within the SCR’s optimal window. This ensures consistently high NO_x_ conversion efficiency, leading to stable NO_x_ emissions that show no significant correlation with VSP fluctuations (Figure 3f). Port operation is mainly distributed within the low-power range (2 to 5 kW/t), featuring frequent transient conditions such as idling, creeping, and gentle acceleration. Here, the China VI DOC+DPF+SCR system exhibits extreme temperature sensitivity, causing substantial NO_x_ emission variations with minor VSP changes. Conversely, freeway driving shifts the VSP distribution to higher levels (2 to 12 kW/t) under steady-state conditions. Despite elevated in-cylinder NO_x_ formation at higher combustion temperatures, the fully activated SCR system with precise urea injection effectively offsets the increased raw emissions, resulting in the observed flat NO_x_ emission.

Using cubic polynomial regression, we established quantitative relationships between VSP and both CO_2_ and NO_x_ emissions, deriving fitted equations for distinct emission categories. Figure 3 demonstrates strong positive correlations between VSP and pollutant emission rates (CO_2_ and NO_x_) across the tested vehicles, with the fitting results showing high goodness-of-fit (R^2^ > 0.8).

### 3.2. Multivariate Coupling Study of Different Influencing Factors and Emissions

#### 3.2.1. Multivariate Correlation of Driving Patterns, Engine Performance, Environmental Conditions and Emissions

**Figure 4:**
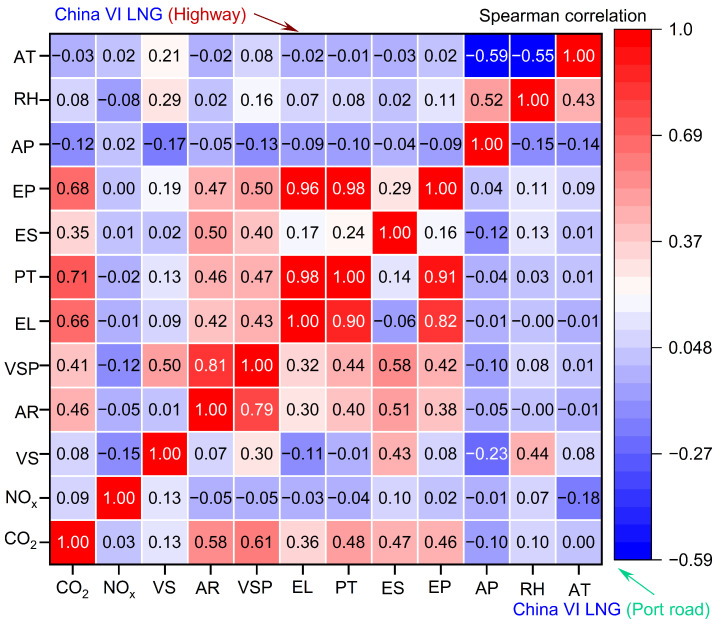
Spearman correlation results for China VI LNG container trucks.

This study utilized two methods, the Spearman correlation coefficient and Random Forest, to investigate the coupling effects of three features on CO_2_ and NO_x_ emission rates. Figure 4 presents the correlation mapping between emissions (CO_2_ and NO_x_) and their determinants in China VI LNG-Powered container trucks. CO_2_ emissions exhibit a positive correlation with engine power, torque percentage, and engine load (r = 0.68, 0.71, and 0.66). On the highway, drivers often increase engine power output and load to maintain or accelerate speed, directly leading to higher fuel consumption for power generation and subsequent CO_2_ emission increases [28]. Conversely, the correlation between CO_2_ emissions and vehicle speed is negligible (r = 0.08). Highway driving typically involves relatively stable speeds, minimizing frequent acceleration/deceleration and thus maintaining consistent fuel consumption and CO_2_ emissions. However, on port road, vehicle speed and VSP have a strong correlation with CO_2_ emissions (r = 0.58 and 0.61). Port roads are typically congested, requiring frequent stops, starts, and accelerations, which lead to the engine operating under unstable load and power output conditions, thereby increasing fuel consumption and CO_2_ emissions. The frequent operational changes on port roads also lead to significant VSP fluctuations, explaining the strong correlation with CO_2_ emissions.

The correlation between NO_x_ emissions and influencing factors is more complex. NO_x_ formation is governed by multiple factors, including combustion temperature, oxygen concentration, and fuel air mixture ratio. For LNG container trucks, although engine parameters (e.g., power and load) can affect combustion conditions, their impact on NO_x_ formation is less direct and pronounced than that on CO_2_ emissions. For the stoichiometric spark-ignition LNG engine equipped with a TWC system, the catalyst provides highly efficient NO_x_ reduction under suitable operating conditions, and the nonlinear catalytic conversion process weakens the direct quantitative relationship between engine operating conditions and tailpipe NO_x_ concentrations [20].

**Figure 5:**
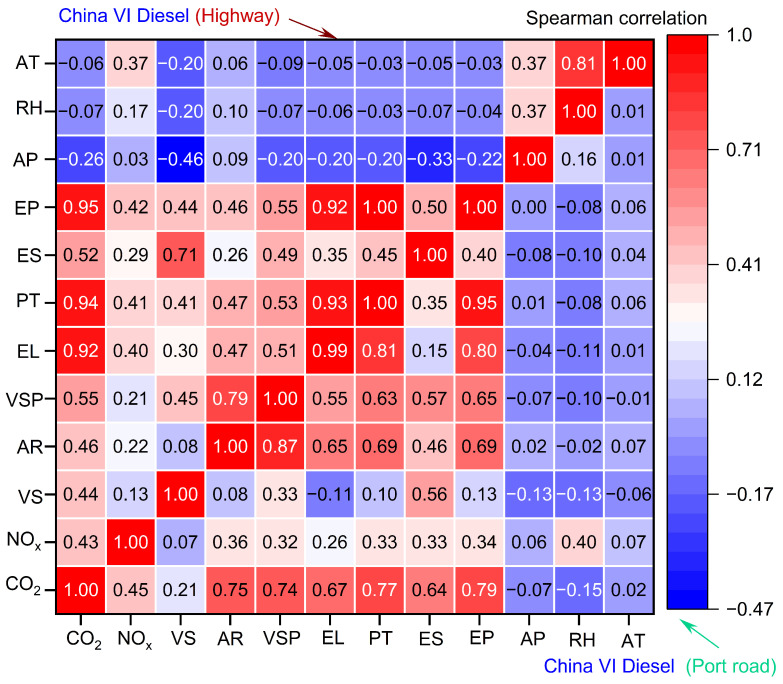
Spearman correlation results for China VI diesel container trucks.

Figure 5 displays the correlation mapping between emissions (CO_2_ and NO_x_) and their determinants in China VI diesel container trucks. Both on highway and port roads, CO_2_ emissions exhibit a strong positive correlation (r = 0.55~0.95) with engine characteristics, including VSP, engine power, and percent torque. In contrast to CO_2_ emissions, the correlation between NO_x_ emissions and the factors listed in the chart is relatively weak. This phenomenon can be ascribed to the intricate characteristics of NO_x_ formation, which is governed by various factors. These include combustion temperature, oxygen concentration, and the fuel air mixture ratio. Furthermore, China VI diesel container trucks are equipped with NO_x_ after-treatment systems (such as SCR systems), which significantly reduce NO_x_ emissions [20]. This mitigation effect obscures the direct correlation between NO_x_ emissions and engine parameters. This mitigating effect weakens the direct relationship between engine operating conditions (e.g., cylinder temperature and exhaust gas recirculation rate) and tailpipe NO_x_ concentrations.

#### 3.2.2. Quantifying Driving, Engine, and External Characteristics Contributions to CO_2_ and NO_x_ Emissions

**Figure 6:**
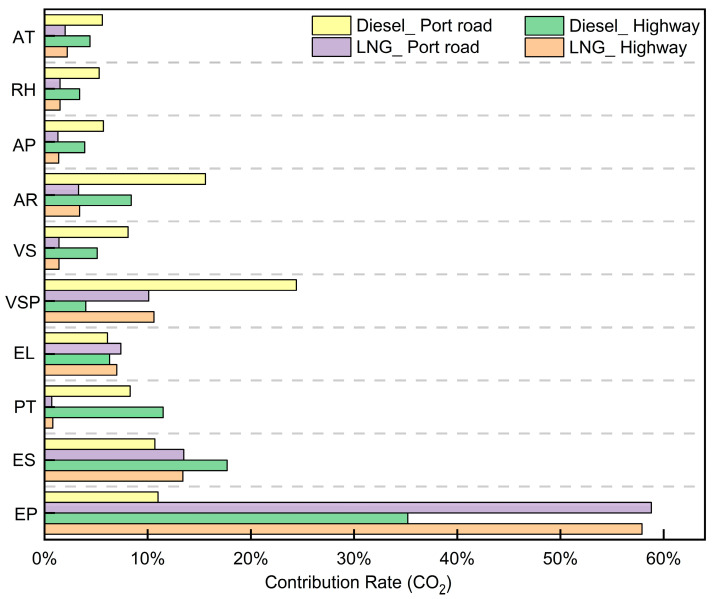
Contribution rates of three characteristics to CO_2_ emissions based on RF.

To determine the contributions of engine, driving, and external characteristics to CO_2_ and NO_x_ emission rates, we applied an RF model. Figure 6 illustrates their respective contributions to CO_2_ emissions. On port roads and highways, environmental parameters (e.g., temperature, humidity, and pressure) generally make a low contribution to CO_2_ emissions from LNG and diesel container trucks. CO_2_ emissions from LNG container trucks are overwhelmingly driven (over 75%) by engine operational characteristics, including load, torque percentage, engine speed, and power. Elevated engine load conditions, characterized by high torque and power output, alter combustion thermodynamics, resulting in degraded efficiency and consequently elevated CO_2_ emissions.

For diesel container trucks, VSP and acceleration are identified as the major factors influencing CO_2_ emission rates, explaining 24% and 16% CO_2_ emissions. Frequent acceleration/deceleration and speed variations elevate engine load and degrade combustion efficiency, directly increasing CO_2_ emissions [13,29].

**Figure 7:**
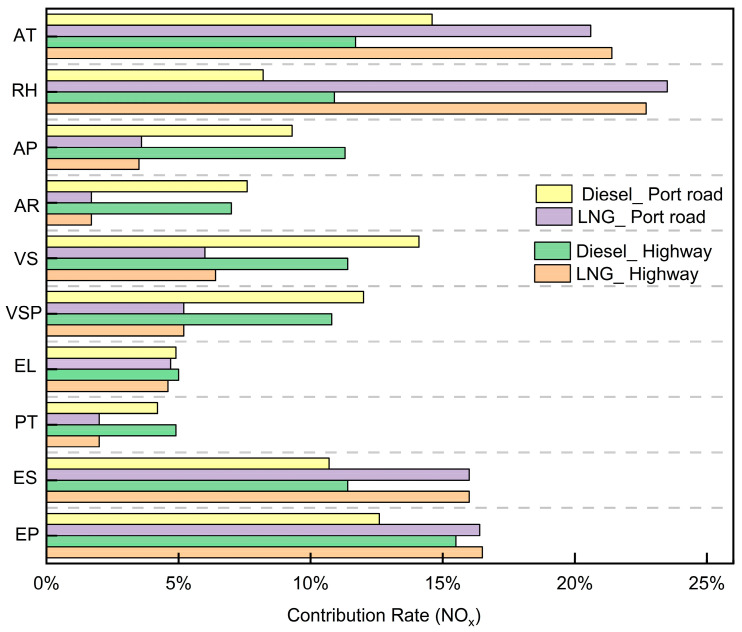
Quantification of the different variables that contribute to NO_x_ emissions.

Figure 7 illustrates the relative contributions of engine, driving, and external factors to NO_x_ emission rates. For China VI LNG container trucks, external environmental factors account for over 48% of NO_x_ emissions. External characteristics such as temperature, humidity, and pressure significantly influence NO_x_ emissions of China VI LNG container trucks, primarily because they directly affect the temperature and pressure within the engine combustion chamber key factors in NO_x_ formation. In contrast, driving and engine characteristics contribute less to NO_x_ emissions, largely due to modern engines incorporating advanced emission control technologies (e.g., SCR systems) and optimized combustion processes, which mitigate NO_x_ production [11,30]. Additionally, the engine management system can further reduce the impact of driving characteristics through real-time adjustments.

For China VI diesel container trucks, driving and engine characteristics contribute more significantly (66%) to NO_x_ emissions, while external factors play a smaller role compared to LNG container trucks. Diesel engines inherently operate at higher combustion temperatures, making ambient temperature changes less impactful on the combustion process than in LNG trucks. Furthermore, China VI vehicle emission standards mandate diesel engines to adopt stricter emission control technologies, which can automatically adjust to varying environmental conditions, thereby minimizing the influence of environmental factors on NO_x_ emissions. For example, SCR systems can adjust urea injection rates based on ambient conditions to maintain stable NO_x_ emissions.

### 3.3. Predicting CO_2_ and NO_x_ Emissions for Different Road Conditions

The Random Forest model was used to predict CO_2_ and NO_x_ emissions, based on an analysis of influencing factors. The model’s effectiveness was assessed through comparison with LR, XGB, GBRT, KNN, and BR models, maintaining identical input parameters across all evaluations [18,31].

**Figure 8:**
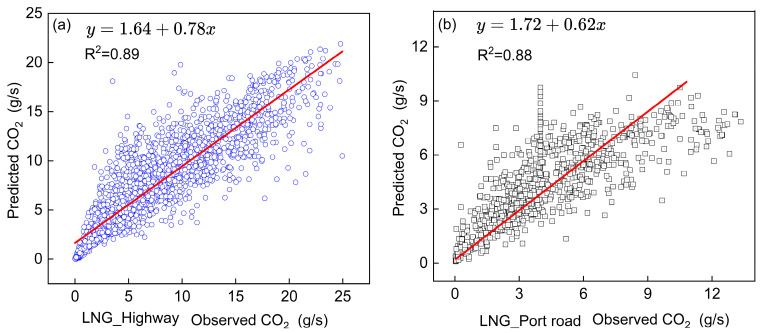
RF model performance for CO_2_ emission prediction in China VI LNG trucks. (**a**) highway; (**b**) port road scenarios (test data).

Overall, the RF model demonstrates superior predictive performance, exhibiting the lowest RMSE and highest R^2^ values among all evaluated models. A case study of CO_2_ emissions from China VI LNG container trucks was conducted to visualize model performance. As shown in Figure 8, the RF model’s predicted CO_2_ values closely match observed values, particularly under highway conditions where it achieves optimal accuracy (RMSE = 0.83, R^2^ = 0.89). Driving patterns on port roads demonstrate greater dynamic complexity relative to highway driving scenarios. Highway driving is characterized by sustained, stable speeds, while port operations feature frequent acceleration–deceleration cycles. These distinct driving regimes result in divergent engine load characteristics and consequent variations in emission generation.

Table 2 quantitatively compares the predictive accuracy of the RF model against baseline algorithms for both CO_2_ and NO_x_ emissions across training and testing datasets. The RF model outperformed the baseline methods in forecasting both pollutants. For example, in the CO_2_ test data sets, RF achieves an 18.8–31.94.63% reduction in RMSE and a 40.8–52.4% improvement in R^2^ relative to the LR model. The RF model demonstrates superior predictive accuracy relative to nonlinear approaches (KNN and BR), achieving RMSE reductions of 1.4–24.0% and R^2^ improvements of 0.8–30.3% for China VI diesel container truck emissions (Table 2).

The RF model exhibits better predictive performance for China VI LNG container trucks than for China VI diesel container trucks (Table 2). For CO_2_, China VI LNG container trucks show a 53.7–68.1% reduction in RMSE and a 1.2–48.2% increase in R^2^ compared to diesel trucks. Analysis of RF–predicted NO_x_ emissions reveals LNG-powered trucks achieve a 25% greater predictive accuracy (R^2^) compared to diesel trucks under port road operating conditions, based on test dataset validation.

### 3.4. Emission Factors of China VI Container Trucks and Comparative Analysis with Previous Studies

#### 3.4.1. CO_2_ Emission Factors

**Figure 9:**
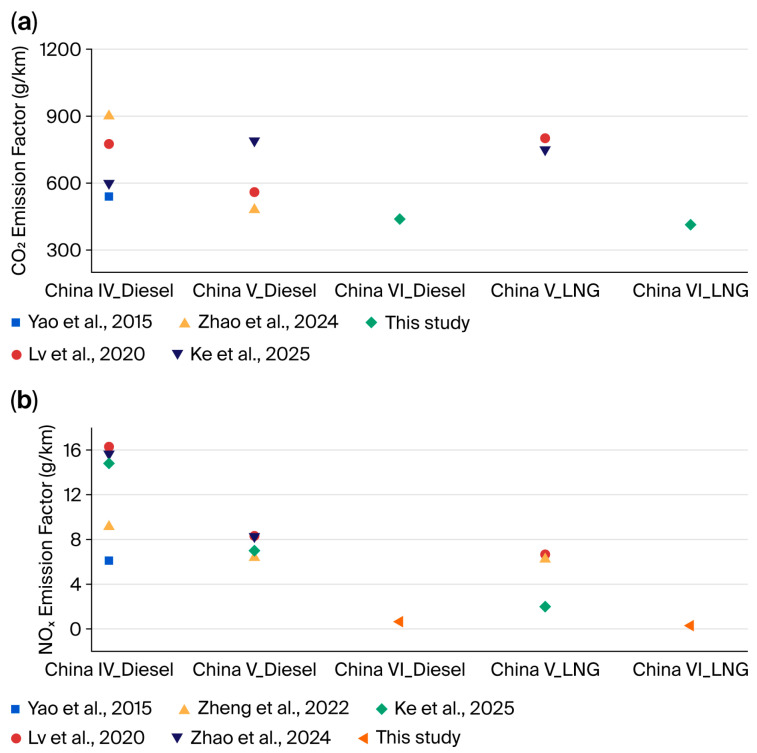
Comparison of the emission factors between this study and previous studies. (**a**) CO_2_ emission factors [2,9,32,33]; (**b**) NO_x_ emission factors [2,9,32,33,34].

To effectively demonstrate the emission levels characteristic of China VI standard vehicles, the specific emission factors of the test vehicle in this study were compared with those of similar vehicle types reported in previous studies [2,9,32,33]. The CO_2_ emission factors of China VI container trucks in this study are presented in Figure 9a (purple diamonds). For China VI diesel vehicles, the measured CO_2_ emission factor was 438.84 g/km, while for China VI LNG vehicles, it was 413.77 g/km. These values are substantially lower than those reported in previous studies for earlier emission standards. For example, Lv et al. (2020) reported CO_2_ emission factors of approximately 559.6 g/km for China V diesel vehicles and 801.17 g/km for China V LNG vehicles [2], while Zhao et al. (2024) reported 480.55 g/km for China V diesel [33]. The observed reduction in CO_2_ emissions for China VI standard vehicles can be primarily attributed to advancements in engine thermal efficiency, which align with the national goals for greenhouse gas mitigation in the transportation sector.

#### 3.4.2. NO_x_ Emission Factors

The NO_x_ emission factors for China VI container trucks in this study (Figure 9b) show a remarkable decline compared to previous standards [2,9,32,33,34]. In this study, the NO_X_ emission factors for the China VI LNG and diesel trucks were 0.29 and 0.65 g/km, respectively. This represents a significant reduction of over 85% compared to China V vehicles. For instance, Zheng et al. (2022) and Ke et al. (2025) reported NO_x_ emission factors of around 7 g/km for China V diesel vehicles [9,34] while Lv et al. (2020) measured values as high as 16 g/km for China IV diesel [2]. For LNG vehicles, the NO_x_ emission factor for China V vehicles was reported to be between 2.5 and 7 g/km (Ke et al., 2025; Lv et al., 2020) [2,9]. The substantial NO_x_ reduction in China VI vehicles is predominantly driven by the mandatory implementation of advanced after-treatment technologies, such as selective catalytic reduction and exhaust gas recirculation, which have been proven to be highly effective in controlling nitrogen oxide emissions.

## 4. Conclusions

A portable emissions measurement system (PEMS)—an integrated platform with gas concentration sensors, vehicle dynamic sensors, and data acquisition modules—was deployed to monitor and compare emissions profiles of China VI LNG and diesel container trucks in both highway and port road operational scenarios. The key conclusions are described below:

Firstly, primary CO_2_ emission hotspots occur during low- to mid-speed acceleration events. On highways, these high-emission zones emerge when speeds surpass 40 km/h concurrent with acceleration exceeding 0.3 m/s^2^, whereas port road emissions exhibit more dispersed spatial distribution patterns.

Secondly, emission rates exhibit a positive correlation with increasing VSP, but the growth rate gradually decelerates. Peak emissions are observed at higher VSP ranges ([10, 12] kW/t) on highways compared to port operations ([4, 5) kW/t). Notably, China VI LNG trucks achieve significantly lower CO_2_ and NO_x_ emission rates. By fitting a third-degree polynomial function, we quantified CO_2_ and NO_x_ emissions across detailed VSP categories and derived fitting equations (R^2^ > 0.8).

Thirdly, distinct emission drivers have different operating environments. On the highway, CO_2_ emissions show a positive correlation with engine power, torque percentage, and engine load (r = 0.68, 0.71, and 0.66), while port road emissions correlate more closely with vehicle speed and VSP (r = 0.58 and 0.61). For LNG trucks, engine characteristics collectively explain over 75% of CO_2_ variability, whereas diesel truck emissions are primarily governed by VSP (24%) and acceleration (16%). Notably, external factors demonstrate reduced influence on NO_x_ emissions in diesel versus LNG trucks under China VI standards.

Finally, the Random Forest model exhibits superior predictive accuracy, particularly under highway conditions where it achieves optimal performance metrics (RMSE = 0.83, R^2^ = 0.89). Comparative analysis reveals significant improvements over Linear Regression, with 18.8–31.9% lower RMSE values and 40.8–52.4% higher R^2^ scores. Furthermore, the model demonstrates enhanced CO_2_ prediction capability for China VI LNG container trucks compared to diesel container trucks.

The findings deliver sensor data-validated parameters for refining emission factor derivation and inventory calculation, with particular relevance to LNG-fueled heavy-duty vehicles. Additionally, they support the advancement of intelligent transportation environmental monitoring technologies integrated with multi-source sensor data. Future research may extend this sensing-based methodology to more heavy-duty vehicle categories and diverse driving scenarios for targeted monitoring applications.

## Figures and Tables

**Table 1:** Description of the experimental vehicle.

Parameter	China VI LNG	China VI Diesel
Curb weight (t)	7.3	8.87
Total traction mass (t)	34.5	40.1
Rated power (kw)	353	420
Idle speed (r/min)	1900	1400
Fuel type	LNG	Diesel
Model year	2023	2020
OBD port	Yes	Yes

**Table 2:** Performance comparison between the RF model and baseline models.

	Items	Model	Train				Test			
			Highway		Port Road		Highway		Port Road	
			RMSE	R^2^	RMSE	R^2^	RMSE	R^2^	RMSE	R^2^
China VI (LNG)	CO_2_	LR	0.92	0.86	0.89	0.86	0.97	0.85	0.94	0.86
		XGB	0.58	0.94	0.55	0.95	0.86	0.88	0.81	0.89
		RF	0.29	0.99	0.28	0.99	0.83	0.89	0.87	0.88
		GBRT	0.67	0.92	0.63	0.93	0.87	0.88	0.83	0.86
		KNN	0.75	0.90	0.70	0.92	1.01	0.84	0.98	0.84
		BR	0.34	0.98	0.34	0.98	0.90	0.87	0.88	0.88
	NO_x_	LR	0.00	0.69	0.01	0.72	0.00	0.68	0.01	0.70
		XGB	0.00	0.87	0.00	0.84	0.00	0.76	0.00	0.84
		RF	0.00	0.97	0.00	0.97	0.00	0.84	0.00	0.97
		GBRT	0.00	0.72	0.00	0.76	0.00	0.64	0.00	0.76
		KNN	0.00	0.73	0.00	0.75	0.00	0.59	0.00	0.75
		BR	0.00	0.96	0.00	0.95	0.00	0.80	0.00	0.95
China VI (Diesel)	CO_2_	LR	3.64	0.68	2.29	0.66	3.67	0.67	2.21	0.66
		XGB	2.01	0.87	1.36	0.89	2.50	0.80	1.81	0.79
		RF	0.90	0.97	0.70	0.94	2.50	0.80	1.79	0.81
		GBRT	2.31	0.83	1.62	0.70	2.61	0.78	1.85	0.67
		KNN	2.65	0.78	1.79	0.73	3.28	0.65	2.08	0.76
		BR	1.16	0.96	0.86	0.92	2.65	0.77	1.90	0.75
	NO_x_	LR	0.00	0.79	0.00	0.78	0.00	0.80	0.00	0.79
		XGB	0.00	0.96	0.00	0.93	0.00	0.91	0.00	0.79
		RF	0.00	0.98	0.00	0.97	0.00	0.90	0.00	0.81
		GBRT	0.00	0.95	0.00	0.89	0.00	0.90	0.00	0.79
		KNN	0.00	0.87	0.00	0.78	0.00	0.82	0.00	0.69
		BR	0.00	0.98	0.00	0.96	0.00	0.89	0.00	0.74

## Data Availability

The datasets analyzed during the current study are available from the corresponding author on reasonable request.

## Abbreviations

The following abbreviations are used in this manuscript:

HDVs	Heavy-duty vehicles
LNG	Liquefied natural gas
PEMS	Portable emissions measurement system
VSP	vehicle-specific power
China VI	China VI vehicle emission standards
OBD	On-Board Diagnostic
NMHC_S_	non-methane hydrocarbons
PM	particulate matter
GHG	Greenhouse gas
TWC	three-way catalyst
DOC	diesel oxidation catalyst
DPF	diesel particulate filter
SCR	selective catalytic reduction
EL	engine load
PT	percentage torque
ES	engine speed
EP	engine power
VS	vehicle speed
VR	vehicle acceleration
AT	ambient temperature
AP	ambient pressure
RH	relative humidity
CV	coefficient of variation
RF	Random Forest
LR	Linear Regression
BR	Bagging Regression
XGB	Extreme Gradient Boosting
GBRT	Gradient Boosted Regression Trees
KNN	k-Nearest Neighbors
RMSE	Root mean square error
